# 1-Laurin-3-Palmitin as a Novel Matrix of Solid Lipid Particles: Higher Loading Capacity of Thymol and Better Stability of Dispersions Than Those of Glyceryl Monostearate and Glyceryl Tripalmitate

**DOI:** 10.3390/nano9040489

**Published:** 2019-03-29

**Authors:** Hao Shi, Shuangshuang Huang, Junbo He, Lijuan Han, Weinong Zhang, Qixin Zhong

**Affiliations:** 1 Key Laboratory for Deep Processing of Major Grain and Oil, Ministry of Education, College of Food Science & Engineering, Wuhan Polytechnic University, Wuhan 430023, China; go_live@yeah.net (H.S.); Huangss0828@163.com (S.H.); hanlj.whpu@hotmail.com (L.H.); 2 Hubei Key Laboratory for Processing and Transformation of Agricultural Products, Wuhan Polytechnic University, Wuhan 430023, China; 3 Department of Food Science, The University of Tennessee, Knoxville, TN 37996, USA; qzhong@utk.edu

**Keywords:** solid lipid nanoparticles, 1-laurin-3-palmitin, thymol, delivery system

## Abstract

To develop solid lipid nanoparticles (SLNs) with a new lipid matrix for delivery of hydrophobic bioactive molecules, high purity 1-laurin-3-palmitin (1,3-LP) was synthesized and the prepared 1,3-LP SLNs were compared with those of two common SLN matrices in glyceryl monostearate (GMS) and glyceryl tripalmitate (PPP). Conditions of preparing SLNs were first optimized by evaluating the particle size, polydispersity index (PDI), zeta-potential, and stability. Thereafter, the performance of SLN loading of a model compound in thymol was studied. The loading capacity of thymol in 1,3-LP SLNs was 16% of lipids and higher than 4% and 12% for GMS- and PPP-SLNs, respectively. The 1,3-LP SLNs also had the best efficiency to entrapment thymol during the prolonged storage. X-ray diffraction (XRD) analyses confirmed the excellent crystalline stability of 1,3-LP leading to the stable entrapment efficiency and better stability of thymol-loaded SLNs. Conversely, the polymorphic transformation of GMS and PPP resulted in the declined entrapment efficiency of thymol in the corresponding SLNs. This work indicated the 1,3-diacylglycerol (DAG) SLNs could be used as a promising delivery system for the encapsulation of hydrophobic bioactive molecules with high loading capacity and stability.

## 1. Introduction

Colloidal delivery systems are frequently studied to encapsulate and control the release of bioactive molecules relevant to drugs, agrochemicals, cosmetics, and food [1,2,3]. These systems include microemulsions [4], nanoemulsions [5], solid lipid nanoparticles (SLNs) [6], nanogels [7], and nanoliposomes [8]. Among them, SLNs were successfully developed in the early 1990s and have shown desirable characteristics as a class of promising delivery systems [9,10,11], such as physical and chemical storage stability, low toxicity, high loading capacity, scalability, and prolonged release of encapsulated compounds [6].

Solid lipids used in SLN formulations include fatty acids [12], triacylglycerols (TAGs) [13,14,15], monoacylglycerols (MAGs) [16,17], and waxes [18,19]. Lipids play an important role in determining the properties of SLNs for delivery of bioactive compounds, such as the loading capacity, entrapment efficiency, stability, particle size, and bioavailability [6]. Lipids with different hydrocarbon chain lengths usually form more lattice defects [6], which favor the loading of lipophilic bioactive molecules. However, the commonly used TAGs, trimyristin and tristearin, and MAGs only have one kind of fatty acid, which would not produce enough lattice defects to contain drugs. Even worse, the crystalline lipid matrices of TAGs and MAGs tend to have the undesired polymorphic transformation from the more disordered and less stable α-form to the more ordered and stable β-form, which reduces the space for the loaded drugs and results in the expulsion of drugs from SLNs [6]. Novel lipid matrices overcoming this disadvantage are significant to advance the science and technology of SLNs.

Diacylglycerols (DAGs), also called diglycerides, have been approved as generally recognized as safe (GRAS) and gained great attention for their unique biological functions [20] such as reducing blood lipids, lowing visceral fat, and preventing weight-related disorders [21]. More importantly, it has been argued that 1,3-DAGs only form stable β-form polymorphic structures [20]. This property may be used to prepare SLNs with a 1,3-DAG core to prevent polymorphic transformation and therefore expulsion of drugs during storage. Additionally, 1,3-DAGs have a polarity between MAGs and TAGs, which may favor the dissolving, loading, and retention of compounds with similar polarity. The molecular structure of 1,3-DAGs can be conveniently tailored to tune the properties of SLNs as delivery systems. However, there are currently no studies on 1,3-DAGs as a sole solid lipid matrix to prepare SLNs.

Thymol (2-isopropyl-5-methylphenol) is the main monoterpene phenol component of essential oil that has been abundantly found in thyme (*Thymus vulgaris*) [22]. It is considered as interesting substance in food industry, not only for its excellent antimicrobial activity against both Gram-positive and Gram-negative bacteria, but also for its GRAS (generally recognized as safe) status [23]. However, the relatively poor water solubility and dispersion homogeneity of thymol may strongly impact the effectiveness against microbials. Nanoencapsulation can greatly improve the physicochemical properties of thymol and improve the antimicrobial effectiveness to inhibit pathogens in food matrices.

The first objective of the present study was to synthesize and characterize high purity 1-laurin-3-palmitin (1,3-LP). The second objective was to study the properties of 1,3-LP as the matrix of SLNs using thymol as a model compound with a medium polarity, with comparison to those prepared with glyceryl monostearate (GMS) and glyceryl tripalmitate (PPP). In addition to loading capacity, entrapment efficiency and entrapment stability of thymol during storage, the polymorphic structures of lipids were studied using X-ray diffraction spectroscopy (XRD) to reveal the structure–function correlations.

## 2. Materials and Methods

### 2.1. Materials

Thymol (>98%), soybean lecithin (>98%), palmitic acid (97%), tetrabutylammonium bromide (97%), Tween 80 (T80), GMS (Analytical Reagent), and PPP (>98%) were purchased from Aladdin Co., Ltd. (Shanghai, China). The 1,3-LP standard was purchased from Shanghai ZZBio. Co., Ltd. (Shanghai, China). All materials were used without further purification.

### 2.2. Synthesis and Characterization of 1,3-LP

**Figure 1:**
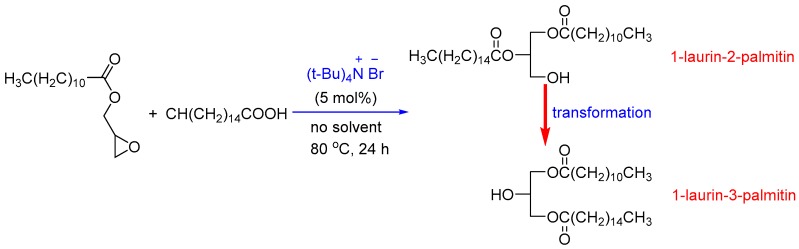
Synthesis route of 1-laurin-3-palmitin (1,3-LP).

The synthesis of 1,3-LP was carried out using the ring opening reaction catalyzed by tetrabutylammonium bromide (TBAB) in the presence of glycidyl laurate, synthesized according to the existing method [24], and palmitic acid (Figure 1) by following the literature method with slight modification [25]. A mixture of glycidyl laurate (10 mmol, 2.56 g), palmitic acid (12 mmol, 3.07 g), and TBAB (0.5 mmol, 0.16 g) was reacted at 100 °C for 24 h. The reaction mixture was then dissolved in n-hexane (40 mL) and washed with water (30 mL, 3 times). After being dried over by Na_2_SO_4_ overnight, the organic solvent was removed by evaporation, and the title compound was subsequently purified by crystallization from methanol and n-hexane (1:3, *v*/*v*), followed by heating in a 55 °C vacuum oven to ensure all the isomer, 1,2-LP, was converted to 1,3-LP.

The melting temperature (Tm) was measured on an X4 microscopic melting point apparatus (Gongyi City Kerui Instrument Co., Ltd., Henan, China) and was uncorrected. Proton nuclear magnetic resonance (^1^H NMR) spectra were recorded at 400 MHz in CDCl_3_ solution on a Varian Mercury Plus 400 spectrometer (Varian, Inc., Palo Alto, CA, USA) and chemical shifts were recorded in parts per million (ppm). The spectra were analyzed using these parameters: *δ* 0.88 (t, 6H, *J* = 8.0 Hz, CH_3_), 1.26 (s, 40 H, CH_2_), 1.63 (s, 4H, CH_2_-CH_2_-CO), 2.33–2.37 (m, 4H, CH_2_-CO), 4.09–4.21 (m, 4H, CH_2_-O-CO). The Fourier transform infrared (FTIR) spectrum of 1,3-LP was collected on a Thermo Nicolet NEXUS 670 FTIR Raman spectrometer (Thermo Nicolet Corp., Madison, WI, USA). The wavenumber range recorded was from 4000 cm^−1^ to 500 cm^−1^ and 16 scans were collected for 1,3-LP. The mass spectrum (MS) of 1,3-LP was obtained on an LTQ Orbitrap XL mass spectrometer (Thermo Fisher Scientific, San Jose, CA, USA). Double distilled water was used throughout the study.

### 2.3. High Performance Liquid Chromatography Coupled with Evaporative Light Scattering Detection (HPLC-ELSD) Analysis of 1,3-LP

A Waters 1525 series HPLC system (Waters Corporation, Milford, MA, USA) and an Alltech 3300 ELSD detector (Alltech Associates, Inc., Deerfield, IL, USA) were used for the HPLC-ELSD analysis. The chromatography separation was performed on an Agilent C18 column (4.6 × 150 mm, 5 μm). An isocratic program was applied with acetonitrile and isopropanol (60:40, *v*/*v*) at a flow rate of 1.0 mL/min and a column temperature of 35 °C. The detection conditions of ELSD were as follows: drift tube temperature of 45 °C, nebulizer gas (N_2_) flow rate of 2.0 L/min, and gain of 4. The injection volume was 20 μL.

### 2.4. Preparation of SLNs

GMS-, 1,3-LP-, and PPP-SLN dispersions were prepared using a microemulsion method [26]. To illustrate the effects of lipid matrix on the thymol-loading performance, all formulations were prepared under the same formulation and conditions. Briefly, 80 mg lipid was melted at 70 °C under continuous stirring (500 rpm). The aqueous phase was prepared by adding soybean lecithin and T80 at a mass ratio of 1:3, 1:2, 1:1, 2:1, and 3:1 to double distilled water (2 g) which was also heated to 70 °C. The mass ratio of lipid to surfactant was fixed at 1:3. Then, the aqueous phase was added rapidly to the lipid phase at 70 °C, after which ethanol, about 0.5 mL, was added dropwise to the pre-emulsion and stirred for 5 min to form a transparent microemulsion. The hot microemulsion was subsequently dispersed in 5 parts of (*v*/*v*) of cold water (2–5 °C). All samples were stored at 25 °C before further analysis.

### 2.5. Determination of Z-Average Mean Diameter, Polydispersity Index (PDI), and Zeta-Potential

The Z-average mean diameter, PDI, and zeta-potential measurements of the prepared SLN dispersions were performed using a Malvern Zetasizer Nano ZS90 Particle Analyzer (Malvern Instruments Ltd., Worcestershire, UK). The laser wavelength was 633 nm, and the material/dispersant reflective index was 1.590/1.330. Samples were prepared by diluting the SLN dispersions eighty-fold using double distilled water at pH 7. Each sample was repeated three times to obtain the mean value.

### 2.6. Comparison of the Loading Capacity and Entrapment Efficiency of SLNs

To verify the drug-loading performance of the three kinds of SLNs, thymol, a natural compound with antimicrobial and antifungal activities [23], was chosen as a model compound. Different amounts of thymol were used to prepare SLN dispersions based on the formula optimized in Section 2.4. The mass ratios of thymol to lipid were set as 4%, 8%, 12%, 16%, 20%, and 24%. The entrapment efficiency (EE) of samples was measured using a Waters 1525 series HPLC system (Waters Corporation, Milford, MA, USA) equipped with a UV-vis detector operating at 230 nm and an Agilent C18 column (4.5 × 250 mm, 5 μm) kept at 25 °C. After filtration of free thymol in SLN dispersions using an ultrafiltration membrane with a molecular weight cut-off of 10,000 Da (Vivaspin^®^, Sartorius, Bagno a Ripoli, Florence, Italy), 20 µL of the permeate was injected to the HPLC. The mobile phase was composed of methanol and deionized water (75:27, *v*/*v*) and running at a flow rate of 1 mL/min during isocratic separation. The amount of encapsulated thymol was calculated based on the total thymol used in encapsulation (Wa) and the free thymol determined in the permeate (Ws). The EE of the SLN dispersions was calculated using the following equation [27]:

EE (%) = [(Wa − Ws)/Wa] × 100(1)

### 2.7. X-ray Powder Diffraction (XRD) Spectroscopy Analysis

XRD was performed by following the literature method with slight modification [28]. In this study, only the spectra of bulk lipids were investigated to understand possible polymorphic structures of lipid matrix in the SLNs during storage. The powders of 1,3-LP, GMS, and PPP were obtained by melting the lipids at 70 °C and cooled immediately at 0–5 °C to simulate the thermal conditions during SLN preparation. The experiments were conducted using a Philips MPD-X’Pert diffractometer (Almelo, The Netherlands) equipped with Cu Kα radiation (λ = 1.54056 Å) and operating at a voltage of 45 kV, a current of 40 mA, and a step width of 0.039°/s over the 2θ range of 5–75°.

### 2.8. Statistical Analysis

The data reported in this paper were presented as mean ± standard deviation (SD). Significance of differences was evaluated using Student’s *t*-test with *p* < 0.05. 

## 3. Results and Discussion

### 3.1. Synthesis of 1,3-LP

**Figure 2:**
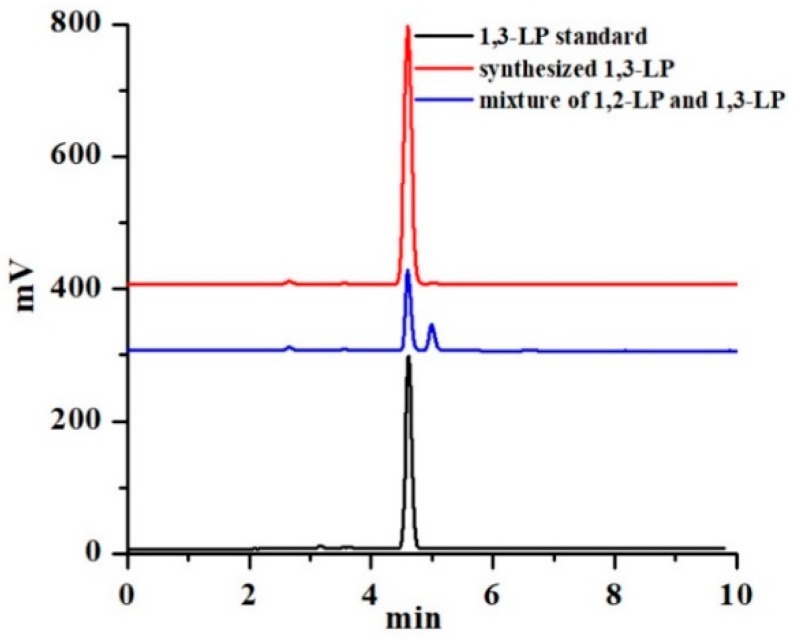
HPLC-ELSD analysis of 1-laurin-3-palmitin (1,3-LP). The peak area ratio of 1,3-LP to 1,2-LP was 7:1 before isomerization. The purity of 1,3-LP was 97% after isomerization.

In HPLC-ELSD, 1,3-LP and 1,2-LP had a retention time of 4.6 min and 5.0 min, respectively (Figure 2), and the ratio of 1,2-LP to 1,3-LP was 7:1 after the ring opening reaction (Figure 1). The purity of 1,3-LP was improved to 97% after isomerization at 55 °C for 72 h, and the total yield was 70%. The final 1,3-LP product appeared as a white solid and had a melting point of 60–62 °C. The present method gives an efficient approach to prepare unsymmetrical 1,3-DAG with good yield and high purity, which can be used as a lipid matrix for fabricating 1,3-DAG SLNs.

**Figure 3:**
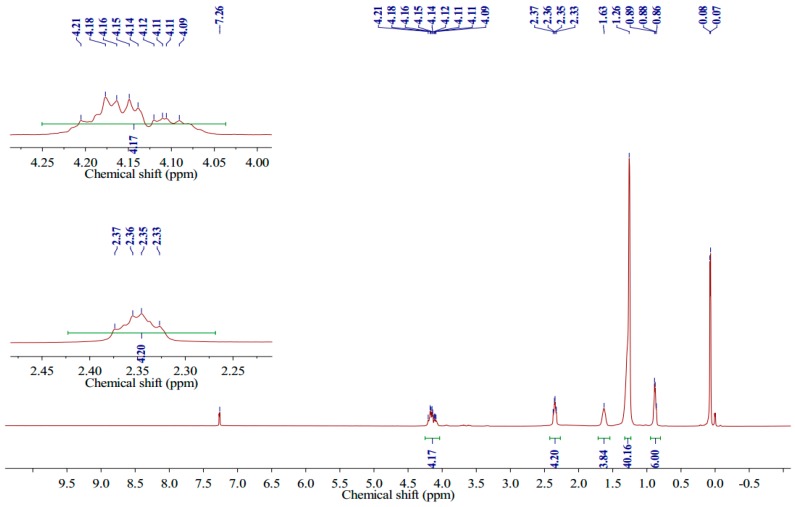
^1^H NMR spectra of synthesized 1,3-LP recorded in CDCl_3_ with Me_4_Si as an internal standard.

**Figure 4:**
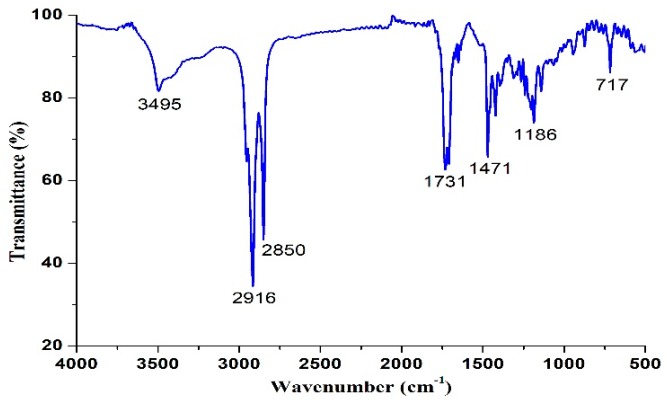
Fourier transform infrared (FTIR) spectrum of 1,3-LP.

**Figure 5:**
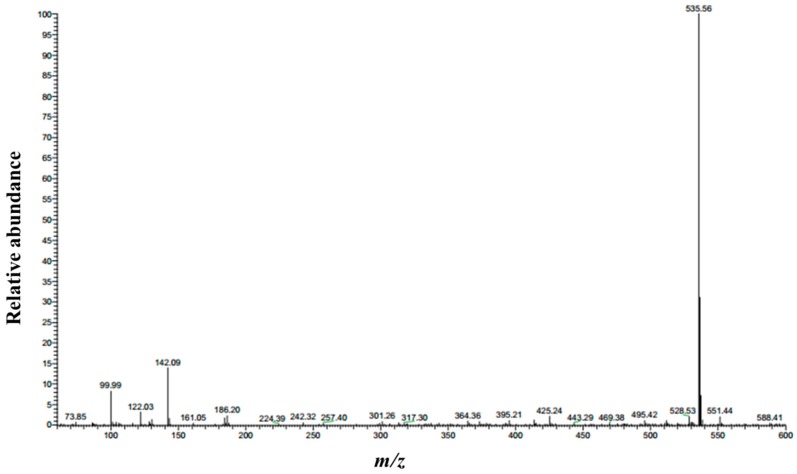
Mass spectrum of 1,3-LP. The [M + Na]^+^ peak was observed at *m*/*z* 535.56.

The structure of 1,3-LP was further confirmed by ^1^H NMR (Figure 3), FTIR (Figure 4), and MS (Figure 5). The ^1^H NMR spectrum of 1,3-LP was in accord with the reported literature [25], showing a triplet for -CH_3_ protons at δ = 0.88, singlet for −CH_2_− protons at δ = 1.26 and 1.63, and multiplet for −CH_2_CO− protons at δ = 2.33–2.37. The two −CH_2_−O−CO− protons also showed multiplets at δ = 4.09–4.21. The FTIR spectrum of 1,3-LP showed specific bands at 3495 cm^−1^ corresponding to stretching of the OH group, 2916 cm^−1^ and 2850 cm^−1^ corresponding to stretching of the alkyl group C−H, 1731 cm^−1^ corresponding to stretching of C=O, and 717 cm^−1^ corresponding to rocking of the long C−H group. The mass spectrum of [M + Na]^+^ of 1,3-LP was observed at *m*/*z* 535.56.

### 3.2. Optimization of Formulations for the Preparation of SLNs

**Figure 6:**
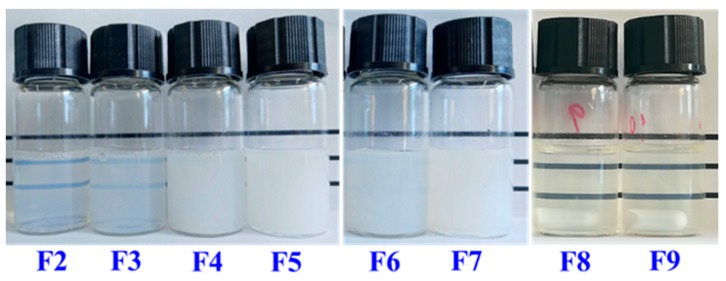
Appearance of freshly prepared glyceryl monostearate- (GMS-), 1,3-LP-, and glyceryl tripalmitate-solid lipid nanoparticle (PPP-SLN) dispersions. F2, F3, F4, F5, F6, F7, F8, and F9 represent the formulations in Table 1.

**Figure 7:**
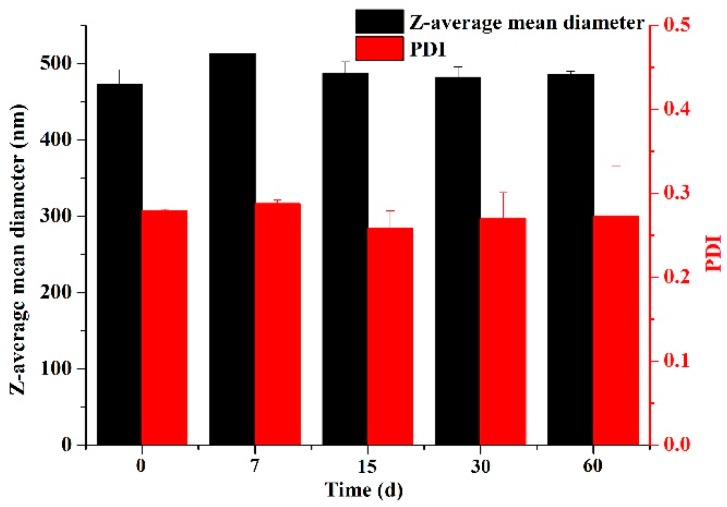
The Z-average mean diameter and polydispersity index (PDI) changes of GMS SLNs prepared using lecithin and T80 at a mass ratio of 3:1 during 60-day storage at 25 °C. Error bars are SD (*n* = 3).

To fabricate stable SLN dispersions, the formulation was optimized firstly using the GMS that has the highest polarity among the three lipids. The surfactants soybean lecithin and T80 (widely utilized to prepare nanoscale lipid structures [29,30,31]) were studied for their mass ratios to prepare stable SLN dispersions. The Z-average mean diameter, PDI, zeta-potential, and visual stability of GMS-SLN dispersions prepared using different mass ratios of lecithin to T80 are summarized in Table 1. With the increase of mass ratio of lecithin/T80, the Z-average mean diameter increased significantly, which agreed with increase in visual turbidity (Figure 6). As lecithin and T80 have an hydrophilic-lipophilic balance (HLB) value of 8.0 and 15.0, respectively, a higher lecithin/T80 mass ratio results in an overall more hydrophobic mixture that is closer to the HLB value of GMS (3.8) and therefore is more effective in reducing the interfacial tension to result in smaller particles [32]. On the contrary, the PDI decreased significantly from 0.39 ± 0.01 to 0.27 ± 0.01 as the lecithin/T80 mass ratio increased from 1:3 to 3:1. This again can be explained by the reduced interfacial tension making it easier to form more homogeneous droplets in microemulsions before quenching to form SLNs. PDI is an important indicator for the homogeneity of particle size distribution, and PDI values below 0.3 indicate the particles are in a narrow size range [33]. Therefore, the lecithin/T80 mass ratios of 2:1 and 3:1 were preferred to obtain relatively uniform GMS SLNs and were used to prepare SLNs using the other two lipids. Characteristics of the 1,3-LP and PPP SLNs are also shown in Table 1. Nanoscale particles and PDI values smaller than 0.3 were observed for both groups of SLNs. It was also observed that the particle dimension decreased with an increase in the number of fatty acid chains on lipids, likely due to the decreased viscosity. For all SLNs prepared at lecithin/T80 mass ratios of 2:1 and 3:1, dispersions were visually stable during two-month storage. These dispersions had a zeta-potential magnitude greater than 20 mV, which is considered to be sufficient to prevent the aggregation of colloidal particles by electrostatic repulsion [34]. The negative zeta-potential results from lecithin and the lecithin/T80 mass ratio of 3:1 were chosen as the optimized formulation for the rest of this study, so as to have a significantly higher magnitude of zeta-potential (Table 1) to stabilize the SLNs in dispersions. For dispersions prepared with a lecithin/T80 mass ratio of 3:1, both the Z-average mean diameter and PDI remained unchanged (*p* > 0.05) over 60-day storage at 25 °C (Figure 7).

### 3.3. Properties of SLNs Loaded with Thymol

Different amounts of thymol were used to prepare SLN dispersions (based on the formulations optimized previously) at a lecithin/T80 mass ratio of 3:1. As the goal was to achieve stable dispersions with PDI smaller than 0.3 (Table 1), the loading capacity of SLNs was considered to be exceeded when dispersions showed visual instability. The loading capacity of thymol in SLNs was 4%, 16%, and 12% of the corresponding lipids when GMS, 1,3-LP, and PPP were studied (Table 2). Thymol is overall hydrophobic but has a polar hydroxyl group. The intermediate polarity of 1,3-LP when compared to GMS and PPP, as discussed previously, may have resulted in the best solubility for thymol to increase the loading capacity. For visually stable dispersions (fresh or after storage), the EE was mostly higher than 95%, with the exception of 85% for the GMS SLNs after storage (Table 2). The data suggest most of the thymol was entrapped in these stable SLNs.

The Z-average mean diameter, PDI, and zeta-potential of treatments are also presented in Table 2. It is evident that the Z-average mean diameter of each group of SLN dispersions increased with the amount of thymol used in the preparation. The HLB of thymol calculated according to Equation (2) is 2.3, which makes the lipid phase more hydrophobic (with a HLB value even smaller than the mixture of lecithin and T80) and therefore results in bigger particles. The increase of the amount of thymol in dispersions also resulted in reduced zeta-potential magnitude and overall poorer stability initially and after storage (Table 2).

HLB = 20 × [M_Hydrophilic_/(M_Hydrophilic_ + M_Lipophilic_)] (2)

where M_Hydrophilic_ and M_Lipophilic_ are the molecular mass of hydrophilic and lipophilic portions of a compound [35].

**Figure 8:**
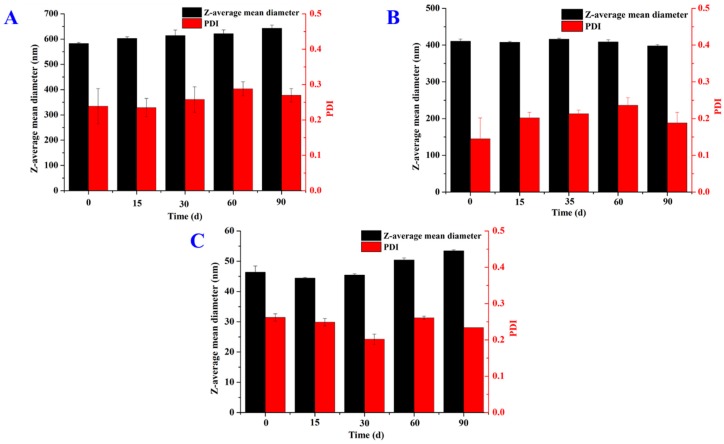
Changes of Z-average mean diameter and PDI of (**A**) GMS SLN dispersions with thymol loaded at 4% mass of lipids, (**B**) 1,3-LP SLN dispersions with thymol loaded at 16% mass of lipids, (**C**) PPP SLN dispersions with thymol loaded at 12% mass of lipids during 90-day storage at 25 °C. Error bars are SD (*n* = 3).

In addition, the Z-average mean diameter at the same amount of thymol used in preparations showed an identical trend to those of bare SLNs (GMS > 1,3-LP > PPP), which is expected due to the minor portion of thymol in the lipid phase. Overall, dispersions with a good initial EE and a PDI smaller than 0.3 maintained good visual stability, which is expected as big particles can phase separate especially after storage. It is worth noting the insignificant changes of Z-average mean diameter and PDI for the 1,3-LP SLN dispersion with thymol being 16% of lipid mass after storage at 25 °C for 90 days (Figure 8B). The same results were found for both the GMS and PPP SLN dispersions with thymol being 4% and 12% of lipid mass, respectively (Figure 8A,C). Considering most thymol remained entrapped (Table 2), SLNs prepared with 1,3-LP showed great promise as delivery systems. The release properties after applying the SLNs (e.g., after dilution with physiological fluid) are to be characterized in the future.

### 3.4. Polymorphic Structures of Lipids Studied with X-ray Powder Diffraction Spectroscopy

**Figure 9:**
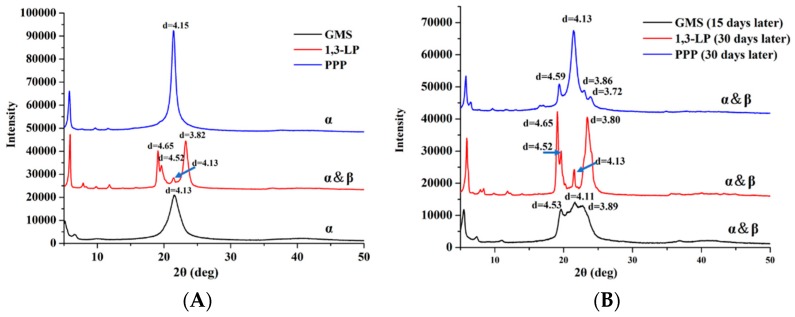
The X-ray powder diffraction (XRD) patterns of GMS, 1,3-LP, and PPP: (**A**) First day of preparation and (**B**) after storage at 25 °C for 15 or 30 days.

Polymorphic structures of the lipid phase play an important role in determining the stability of SLNs and the encapsulated compounds [6]. The XRD spectra of bulk GMS, 1,3-LP, and PPP following thermal treatments analogous to SLN preparation and storage at 25 °C were characterized, shown in Figure 9. According to the XRD American Oil Chemists’ Society (AOCS) method [36], the α-form polymorph displays as one peak at a d-spacing of 4.1 Å, while the β-form displays as two peaks at a d-spacing of 4.6 Å and 3.8 Å. For the freshly prepared lipid crystals, the diffraction patterns in Figure 9A showed GMS and PPP both had one single high intensity peak near the d-spacing of 4.15 Å, which verified the dominant presence of α-form crystals in these two lipids. In contrast, the diffraction peaks of 1,3-LP were mainly located at d-spacings of 4.65 Å, 4.52 Å and 3.82 Å, indicating the formation of β-form polymorph crystals, and, a small peak at a d-spacing of 4.13 Å had a small portion of α-form crystals.

Due to the instability of the hexagonal (α-form polymorph) arrangement of lipid molecules, the polymorphic structures of GMS and PPP crystals changed after 15- and 30-day storage at 25 °C (Figure 9B), respectively. For GMS, almost one-half of the α-form polymorph transformed into the β-form, which can explain the reduction of EE (expulsion of thymol) and phase separation of dispersions (Table 2). For PPP, the newly formed diffraction peaks located at d-spacings of 4.59 Å, 3.86 Å, and 3.72 Å are in accordance with a previous report [37] and demonstrated the formation of β-form crystals from the α-form. The extent of polymorphic transformation in PPP crystals is less than that of GMS, which agrees with changes in thymol EE and visual stability after storage (Table 2). The diffraction patterns of 1,3-LP did not display significant variations after 30-day storage at 25 °C, which agrees with the excellent stability and maintained EE of dispersions prepared with thymol at a level up to 16% of 1,3-LP (Table 2). Furthermore, as the d-spacing obtained from the XRD reflects the subcell dimension within the crystal lattice [38], the high d-spacing of the triclinic arrangement of the β-form polymorph can provide more space for the encapsulation of bioactive molecules. Thereby, the 1,3-LP SLNs, with dominant existence in β-form polymorphic crystals, had a higher thymol loading capacity than SLNs prepared with GMS and PPP (Table 2).

## 4. Conclusions

In this work, the synthesis of high purity 1,3-LP was achieved for use as a solid lipid matrix to prepare SLNs. The stable SLN dispersions of 1,3-LP, GMS, and PPP were optimized at a higher lecithin/T80 mass ratio (3:1), as a result of the closer matching of lipid HLB and surfactant mixture HLB during the preparation of microemulsions. The intermediate polarity of 1,3-LP enabled a higher thymol loading capacity in SLNs than the other two lipids and an EE of >99%. Contrasting with GMS and PPP with thermodynamically unstable α-form crystals and transformation to β-form after storage, the dominance of the β-form polymorph in initial 1,3-LP crystals suggested that the lipid matrix of 1,3-LP not only enhanced the loading of thymol in SLNs but also prevented polymorphic transformation to preserve the stability of SLNs and entrapment efficiency of thymol during storage. The findings in this work suggest for the first time that 1,3-DAG can be used to fabricate a new class of stable SLNs as a delivery system for lipophilic bioactive molecules to overcome challenges of SLNs prepared with conventional MAGs and TAGs.

## Figures and Tables

**Table 1:** The Z-average mean diameter (Z-average), PDI, zeta-potential (Zeta), and storage stability (at 25 °C for up to two months) of SLN dispersions prepared with three types of lipids using different mass ratios of lecithin and T80. All samples were prepared with 80 mg lipid, 240 mg surfactants, 2 g double distilled water, and 0.5 mL ethanol.

Lipid	F ^1^	Lecithin/T80	Z-Average (nm)	PDI	Zeta (mV) ^2^	Visual Stability
GMS	F1	1:3	91 ± 4	0.39 ± 0.01	ND	Precipitate
	F2	1:2	178 ± 3	0.32 ± 0.03	ND	Stable for 2 weeks
	F3	1:1	290 ± 8	0.30 ± 0.01	ND	Stable for 1 month
	F4	2:1	425 ± 8	0.28 ± 0.01	−25.60 ± 2.50	Stable for 2 months
	F5	3:1	473 ± 19	0.27 ± 0.01	−34.10 ± 2.00	Stable for 2 months
1,3-LP	F6	2:1	300 ± 5	0.26 ± 0.01	−24.50 ± 1.42	Stable for 2 months
	F7	3:1	327 ± 4	0.28 ± 0.01	−29.50 ± 1.85	Stable for 2 months
PPP	F8	2:1	38 ± 2	0.25 ± 0.02	−22.00 ± 2.91	Stable for 2 months
	F9	3:1	38 ± 1	0.26 ± 0.01	−24.50 ± 1.51	Stable for 2 months

^1^ F, Formulation. ^2^ ND, not determined due to poor stability of dispersions.

**Table 2:** The Z-average mean diameter (Z-average), PDI, zeta-potential (Zeta), and encapsulation efficiency (EE%) for the thymol loaded GMS-, 1,3-LP-, and PPP-SLN.

Lipid	F ^1^	Thymol/Lipid (%)	Z-Average (nm)	PDI	Zeta (mV)	EE (%) ^2^	
						Day 0	Day 60
GMS	F10	4	582 ± 5	0.24 ± 0.05	−14.30 ± 0.25	99	85
	F11	8	591 ± 4	0.27 ± 0.01	−16.50 ± 1.15	99	ND
	F12	12	620 ± 14	0.26 ± 0.02	−12.60 ± 0.45	99	ND
	F13	16	675 ± 16	0.33 ± 0.03	−15.60 ± 0.55	ND	ND
1,3-LP	F14	4	265 ± 1	0.28 ± 0.01	−14.20 ± 0.40	>99/	>99
	F15	8	311 ± 3	0.22 ± 0.02	−17.80 ± 1.22	>99/	>99
	F16	12	379 ± 5	0.26 ± 0.01	−14.60± 2.59	>99/	>99
	F17	16	410 ± 6	0.15 ± 0.06	−16.00 ± 0.20	>99	>99
	F18	20	476 ± 7	0.22 ± 0.01	−17.60 ± 0.50	99	ND
	F19	24	510 ± 6	0.28 ± 0.03	−18.20 ± 1.17	98	ND
PPP	F20	4	31 ± 0	0.15 ± 0.01	−11.70 ± 0.95	>99	>99
	F21	8	39 ± 2	0.25 ± 0.01	−13.30 ± 0.78	>99	98
	F22	12	46 ± 2	0.26 ± 0.01	−13.00 ± 0.55	>99	95
	F23	16	58 ± 4	0.32 ± 0.05	−15.90 ± 1.05	ND	ND
	F24	20	97 ± 1	0.41 ± 0.01	−18.50 ± 0.36	ND	ND
	F25	24	94 ± 1	0.79 ± 0.01	−26.60 ± 1.67	ND	ND

^1^ F, Formulation. ^2^ ND, not determined as phase separation was observed.
